# Vocal Cord Palsy as a Complication of Epidural Anaesthesia

**DOI:** 10.1155/2018/6543656

**Published:** 2018-10-25

**Authors:** Laura Mc Loughlin, Orla Young

**Affiliations:** Department of Otorhinolaryngology Head & Neck Surgery, Galway University Hospital, Galway, Ireland

## Abstract

Cranial nerve palsy is a rare but recognised complication of epidural anaesthesia, most commonly presenting as diplopia secondary to abducens nerve palsy. While upper cranial nerve palsies have been documented on numerous occasions, lower cranial nerve palsies, including recurrent laryngeal nerve palsy, are exceedingly rare. This case describes a 37-year-old female who, following epidural anaesthesia for spontaneous vaginal delivery of her first child, presented with dysphonia. Flexible laryngoscopy confirmed a left vocal cord palsy, and computed tomography ruled out any mass lesions along the course of the recurrent laryngeal nerve. Here, we discuss a case of vocal cord palsy secondary to epidural anaesthesia, an extremely rare complication. We also discuss the proposed etiology, treatment, and outcomes in patients with this condition. Cranial nerve palsy should be an important differential in patients presenting with dysphonia following spinal or epidural anaesthesia.

## 1. Introduction

Epidural block is a commonly used method of analgesia in obstetrics and surgery of the lower abdomen and extremities. Cranial nerve palsy is an uncommon but recognised complication following spinal and epidural anaesthesia. The current incidence is relatively unknown, with older studies reporting an incidence of 1 in 200 to 1 in 1200 cases [1]. This is likely an overestimate, given the advances in spinal and epidural analgesia in recent decades. Oculomotor, trochlear, trigeminal, abducens, facial, and vestibulocochlear nerve palsies are all recognised complications following spinal and epidural anaesthesia. The abducens nerve is most commonly affected, with patients generally presenting with diplopia. Lower cranial nerve palsies such as vagus nerve palsy are much less well recognised, with only five previously reported cases to our knowledge.

## 2. Case Report

A 37-year-old female teacher presented to the ENT clinic with a four-month history of hoarseness and difficulty in voice production. Her background history was significant only for a recent diagnosis of mild hemochromatosis, and she was on no regular medication.

Four months before, she had delivered a healthy baby boy via normal vaginal delivery with epidural analgesia. The delivery was uneventful, aside from issues with assymetrical epidural block requiring manipulation. She did not suffer with postdural puncture headache, and no drops in blood pressure were noted. Immediately postpartum, she noticed marked hoarseness and had difficulty in voice production. She denied any sore throat, dysphagia, or choking episodes.

On examination, she was markedly hoarse, and flexible laryngoscopy revealed left-sided vocal cord paralysis with no evidence of any mass lesions.

Her neurological exam was otherwise normal.

Computed tomography (CT) of the neck and thorax was performed which showed no lesions along the course of the recurrent laryngeal nerve.

She was advised to rest her voice and delay her return to work. She was not prescribed any medication.

On subsequent review the following month, her voice showed marginal improvement; however, on flexible laryngoscopy, the left vocal cord remained paralysed. Two months later, movement had begun to recover, with complete closure of the vocal cords on maximal strain.

By nine months postpartum, her voice had returned to normal, with flexible laryngoscopy demonstrating full return to movement of the left vocal cord.

## 3. Discussion

Vocal cord paresis results from injury to the ipsilateral recurrent laryngeal nerve, a branch of the vagus, at any point along its course, with resulting dysphonia. A 15-year retrospective study by Takano et al. [2] showed that the majority of vocal cord palsies were as a result of iatrogenic injury to the recurrent laryngeal nerve (58.5%), most commonly following thyroid and cardiothoracic surgery. Vocal cord paresis may also occur as a result of malignant disease, especially lung and thyroid malignancy, causing invasion of the nerve. Up to one-third of cases are reported as idiopathic, in which identifiable causes have been excluded [2, 3]. Other less common causes of acquired recurrent laryngeal nerve injury include cerebrovascular disease, infections such as tuberculosis, direct laryngeal trauma, and neurological disorders such as multiple sclerosis [2, 3].

While there have been numerous reports of upper cranial nerve palsies associated with spinal and epidural anaesthesia, lower cranial nerve palsies including vagal and recurrent laryngeal nerve palsies are much less documented. Oculomotor, trochlear, trigeminal, abducens, facial, and vestibulocochlear nerve palsies are well-recognised complications with the abducens nerve being the most commonly affected one. To date, only five cases of vocal cord paralysis following spinal or epidural anaesthesia have been documented to our knowledge. Two have occurred in the obstetric population, one following epidural anaesthesia and one following combined spinal epidural anaesthesia. The others have occurred in the orthopedic population, all following spinal anaesthesia. In each case, the vocal cord palsy was transient, lasting between 8 weeks and 1 year.

Table 1 describes the previous reported cases of unilateral vocal cord palsy secondary to spinal or epidural anaesthesia.

In their case series, Guardiani et al. [4] suggested that traction on the vagus nerve secondary to intracranial hypotension was the cause for development of cranial nerve palsy. One of their patients underwent laryngeal electromyography showing denervation of the cricothyroid muscle supplied by the superior laryngeal nerve, indicating a vagal neuropathy, supporting their hypothesis. This suggested mechanism is similar to the proposed pathophysiology of postdural puncture headache, thought to be due to intracranial hypotension secondary to loss of CSF volume. Indeed, in a review [7] of 43 instances of cranial nerve palsy following spinal and epidural anaesthesia in obstetric practice, 27 cases were associated with postdural puncture headache, suggesting a common causality between the two. The predilection for abducens nerve injury would also support this hypothesis, with the long intracranial course of the nerve particularly vulnerable to traction injury.

Current accepted treatment options for unilateral vocal cord paralysis consist of observation, speech therapy, or surgery [8]. The majority of identified cases, including our own, have been treated conservatively. In one case, medialisation of the vocal cord with methylcellulose was performed for good effect; however, it does not seem to have had an overall effect on the length of time taken for the palsy to resolve, when compared with the other cases.

In each of the cases, the palsy resolved without intervention, leading us to believe that these neuropathies are ultimately self-limiting in nature.

## 4. Conclusion

Vocal cord palsy may occur as a complication of spinal and epidural anaesthesia and should be considered in cases where dysphonia occurs shortly following such procedures. They are usually transient in nature, lasting between a number of weeks to a year. It is important to be cognisant of vocal cord palsy as a complication of spinal and epidural anaesthesia and consider it as a differential in the absence of other pathologies.

## Figures and Tables

**Table 1:** Summary of reported cases of unilateral vocal cord palsy following spinal or epidural anaesthesia.

Case	Age	Gender	Type of anaesthesia	Procedure	Side of paresis	Timing of onset	Duration	Treatment
Guardiani et al. [4]	50	F	Spinal	Knee arthroplasty	Right	Immediate	1 year	Medialisation with methylcellulose
Guardiani et al. [4]	60	F	Spinal	Knee arthroplasty	Left	4 days	1 year	Observation
Guardiani et al. [4]	30	F	Combined spinal epidural	Vaginal delivery	Right (1st)/left (recurrent)	1 week/3 days	6 months	Tapering dose steroids
Perez et al. [5]	30	F	Epidural	Caesarian section	Right	3 days	6 months	Observation
Guevara et al. [6]	47	F	Spinal	ORIF tibia and fibula	Right	1 day	8 weeks	Observation
